# Comparison of five methods for genomic breeding value estimation for the common dataset of the 15^th ^QTL-MAS Workshop

**DOI:** 10.1186/1753-6561-6-S2-S13

**Published:** 2012-05-21

**Authors:** Chong-Long Wang, Pei-Pei Ma, Zhe Zhang, Xiang-Dong Ding, Jian-Feng Liu, Wei-Xuan Fu, Zi-Qing Weng, Qin Zhang

**Affiliations:** 1 Key Laboratory of Animal Genetics, Breeding and Reproduction, Ministry of Agriculture of China, College of Animal Science and Technology, China Agricultural University, Beijing 100193, China; 2 Institute of Animal Husbandry and Veterinary Medicine, Anhui Academy of Agricultural Sciences, Hefei 230031, China

## Abstract

**Background:**

Genomic breeding value estimation is the key step in genomic selection. Among many approaches, BLUP methods and Bayesian methods are most commonly used for estimating genomic breeding values. Here, we applied two BLUP methods, TABLUP and GBLUP, and three Bayesian methods, BayesA, BayesB and BayesCπ, to the common dataset provided by the 15^th ^QTL-MAS Workshop to evaluate and compare their predictive performances.

**Results:**

For the 1000 progenies without phenotypic values, the correlations between GEBVs by different methods ranged from 0.812 (GBLUP and BayesCπ) to 0.997 (TABLUP and BayesB). The accuracies of GEBVs (measured as correlations between true breeding values (TBVs) and GEBVs) were from 0.774 (GBLUP) to 0.938 (BayesCπ) and the biases of GEBVs (measure as regressions of TBVs on GEBVs) were from 1.033 (TABLUP) to 1.648 (GBLUP). The three Bayesian methods and TABLUP had similar accuracy and bias.

**Conclusions:**

BayesA, BayesB, BayesCπ and TABLUP performed similarly and satisfactorily and remarkably outperformed GBLUP for genomic breeding value estimation in this dataset. TABLUP is a promising method for genomic breeding value estimation because of its easy computation of reliabilities of GEBVs and its easy extension to real life conditions such as multiple traits and consideration of individuals without genotypes.

## Background

The goal of genomic selection (GS) [1] is to capture all quantitative trait loci (QTL) influencing a trait by tracing all chromosome segments defined by adjacent markers. With use of highly dense markers, GS is supposed to be able to overcome the problem of traditional maker assisted selection (MAS) that only a limited proportion of the total genetic variance is captured by the markers of QTL. GS has become feasible very recently with the high throughput genotyping technology and the availability of highly dense markers covering whole genome. Genomic breeding value estimation is the key step in GS. A number of approaches have been proposed for estimating genomic breeding values [1-9], among which BLUP methods and Bayesian methods are most commonly used. Here, we applied two BLUP methods (GBLUP [3], TABLUP [4]) and three Bayesian methods (BayesA, BayesB [1], BayesCπ [5]) to the common dataset provided by the 15^th ^QTL-MAS Workshop to evaluate and compare their predictive performances.

## Methods

### Dataset

The common dataset consisted of an outbred population, which had been simulated using the LDSO software [10], with 1000 generations of 1000 individuals, followed by 30 generations of 150 individuals. 9990 SNP markers were distributed on 5 chromosomes. Each chromosome had a size of 1 Morgan and carried 1998 evenly distributed SNPs (1 SNP every 0.05 cM).

The final dataset used for evaluating genomic selection consisted of 3220 individuals, including 20 sires, 200 dams (each sire mated with 10 dams) and 3000 progenies (15 per dam). All individuals were genotyped for the 9990 SNPs without missing or genotyping error. Of the 15 progenies of each dam, 10 were phenotyped for a continuous trait. The 2000 progenies with phenotypic records and the other 1000 individuals (which had simulated true breeding values) without phenotypic records were treated as reference and validation population, respectively.

### Estimation of variance components and EBVs

The variance components and the traditional BLUP EBVs were estimated using phenotypes and pedigree and the software DMUv6 [11] based on the following model:

y=1μ+Za+e

where **y **is the vector of phenotypes of individuals in the reference population, *μ *is the overall mean, **a **is the vector of additive genetic effects of the phenotyped individuals and their parents, **Z **is the incidence matrix of **a**, and **e **is the vector of residual errors. The variance-covariance matrices of **a **and **e **are $A\sigma_{a}^{2}$ and $I\sigma_{e}^{2}$, respectively, where A is the additive genetic relationship matrix, $\sigma_{a}^{2}$ is the additive genetic variance, and $\sigma_{e}^{2}$ is the residual variance.

The reliabilities of the traditional EBVs were obtained from DMU directly and calculated as the square of the correlation between EBVs and the true unknown breeding values.

### Estimation of SNP effects

BayesA, BayesB and BayesCπ were used to estimate SNP effects in the reference population based on the following model:

y=1μ+Xg+e

where **g **is the vector of random SNP effects, **X **is the matrix of genotype indicators (with values 0, 1, or 2 for genotypes 11, 12, and 22, respectively).

The differences between the three Bayesian methods lay in the assumptions for the prior distribution of SNP effects. BayesA assumes that all SNPs have an effect, but each has a different variance. BayesB and BayesCπ assume that each SNP has either an effect of zero or non-zero with probabilities π and 1-π, respectively, and for those having non-zero effects it is assumed that each SNP has a different variance in BayesB and a common variance in BayesCπ. In addition, in BayesB π is treated as a known parameter, while in BayesCπ it is treated as an unknown parameter with a uniform (0, 1) prior distribution. In this study, we set π = 0.99 for BayesB, and adopted the same prior distributions of **g **and **e **for the three Bayesian methods as those in [1,5].

The Markov chain was run for 50,000 cycles of Gibbs sampling (for BayesB, 100 additional cycles of Metropolis-Hastings sampling were performed for the SNP effect variance in each Gibbs sampling cycle), and the first 5000 cycles were discarded as burn-in. All the samples of SNP effects after burn-in were averaged to obtain the SNP effect estimate.

### Calculation of GEBVs

The genomic estimated breeding values (GEBVs) of all genotyped individuals were obtained using five methods: BayesA, BayesB, BayesCπ, GBLUP and TABLUP.

For BayesA, BayesB and BayesCπ, the GEBV of a genotyped individual was calculated as the sum of all marker effects according to its marker genotypes [1].

For GBLUP and TABLUP, the GEBVs were estimated based on the following model:

y=1μ+Zu+e

where **u **is the vector of genomic breeding values of all genotyped individuals with the variance-covariance matrix equal to $G\sigma_{u}^{2}$ for GBLUP or $TA\sigma_{u}^{2}$ for TABLUP. $\sigma_{u}^{2}$ is the additive genetic variance estimated from the reference population.

The **G **matrix (realized relationship matrix) was constructed by using genotypes of all markers [3]. The **TA **matrix (trait-specific marker-derived relationship matrix), was constructed by using genotypes of all markers with each marker being weighted with its estimated effect obtained from BayesB following the rules proposed by Zhang et al. [4].

The accuracies of GEBVs were calculated as the correlation between GEBVs and the simulated true breeding values.

## Results and discussion

### Variance components

The estimated additive genetic variance and residual variance were 24.82 and 58.65, respectively. Therefore, the estimated heritability was 0.30. These estimates were used for the subsequent estimation of SNP effects and GEBVs.

### Estimates of SNP effects

**Figure 1:**
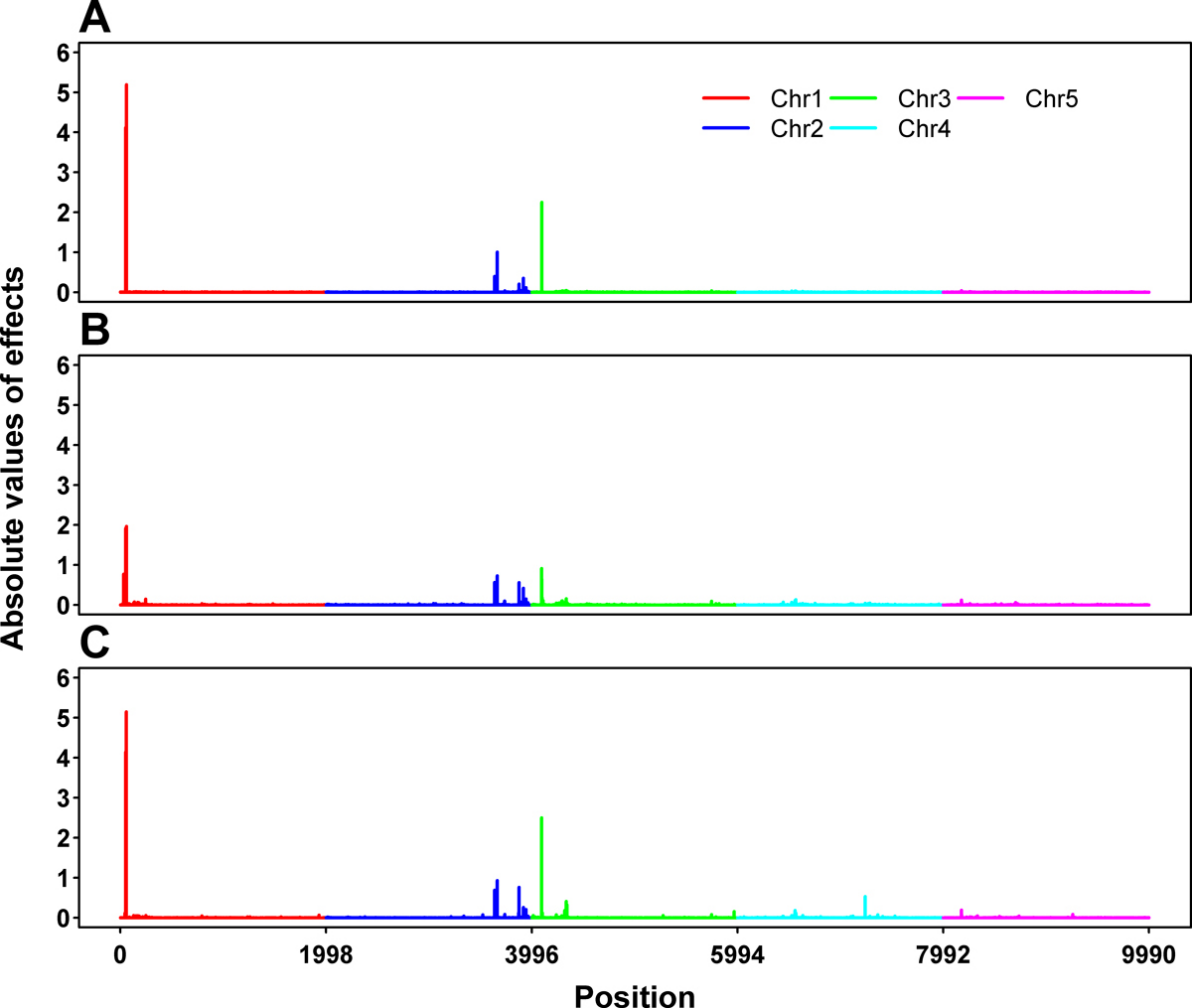
**Absolute values of estimated SNP effects by BayesA (A), BayesB (B) and BayesCπ (C)**.

Figure 1 includes the profiles of SNP effects estimated by BayesA (Figure 1A), BayesB (Figure 1B) and BayesCπ (Figure 1C). These estimated effects, which are obviously not evenly distributed, reflect the underlying architecture of the trait. The estimated value of π in BayesCπ is 0.9986. In general, the SNP effect profiles from the three Bayesian methods are quite similar. In particular, all of the three methods show a big peak on chromosome 1, two peaks on chromosome 2, and a peak on chromosome 3. In addition, BayesCπ shows another peak on chromosome 3 and a peak on chromosome 4. No peaks appear on chromosome 5 for all of the three methods. The peak positions and the corresponding SNP effect estimates are given in Table 1. For chromosomes 1, 2 and 3, where one, two and two additive QTL were simulated, respectively, these peak positions match all the simulated QTL positions quite well, except that BayesA and BayesB missed one QTL on chromosome 3. For chromosomes 4 and 5, where an imprinted QTL and two epistatic QTL were simulated, respectively, either no peak was detected or the detected peak is far away from the simulated position. From these results, it seems that these methods could also serve as tools for QTL mapping and BayesCπ performed better in this respect. The drawback of BayesA and BayesB regarding the impact of prior hyperparameters and treating the prior probability π as known has been addressed by Gianola et al. [12] and Habier et al. [5]. Our results partially confirmed their arguments.

**Table 1:** Peak positions of profiles of the estimated SNP effects and the corresponding estimated SNP effects

Method	Chr. 1		Chr. 2		Chr. 3		Chr. 4	
	**Pos**.	Effect	**Pos**.	Effect	**Pos**.	Effect	**Pos**.	Effect
BayesA	59	5.19±0.37	3660	1.01±0.90	4094	2.25±0.40		
			3914	0.35±0.73				
BayesB	59	1.96±2.13	3660	0.73±0.82	4092	0.91±1.17		
			3873	0.56±0.65				
BayesCπ	58	5.15±0.42	3660	0.93±0.96	4092	2.50±0.76	7234	0.53±1.51
			3873	0.76±0.75	4331	0.41±0.67		
Simulated QTL	57		3638		4100		6644	
			3875		4300			

### Correlations between GEBVs by different methods and between EBVs and GEBVs for the 20 sires

For the 20 sires, the reliability of traditional EBVs was 0.95. Table 2 shows the correlations between GEBVs by different methods and between EBVs and GEBVs of the 20 sires. The correlations between EBVs and GEBVs by different methods ranged from 0.933 to 0.966, and the highest correlation was given by GBLUP and the lowest by BayesCπ. In general, the GEBVs by different methods were highly correlated with the correlation coefficients over 0.95, indicating that the GEBVs for the 20 sires by different methods were quite consistent.

**Table 2:** Correlations between GEBVs by different methods (the first 4 columns) and between traditional EBVs and GEBVs (the last column) for the 20 sires

	BayesB	BayesCπ	TABLUP	GBLUP	Traditional EBV
BayesA	0.999	0.995	0.995	0.972	0.942
BayesB		0.992	0.998	0.978	0.947
BayesCπ			0.986	0.956	0.933
TABLUP				0.986	0.952
GBLUP					0.966

### Correlations between GEBVs by different methods for the 1000 progenies without phenotypic values

Table 3 shows the correlations between GEBVs by different methods for the 1000 progenies without phenotypic values. The correlations ranged from 0.812 to 0.997, and the highest correlation was between TABLUP and BayesB, and the lowest between GBLUP and BayesCπ. The correlations among the three Bayesian methods and TABLUP are all very high (over 0.97), indicating high similarity in GEBVs from these methods, while the correlations between them and GBLUP are all less than 0.9, indicating some differences in GEBVs exist herein.

**Table 3:** Correlations between GEBVs by different methods for the 1000 progenies without phenotypic values.

	BayesB	BayesCπ	TABLUP	GBLUP
BayesA	0.991	0.985	0.983	0.841
BayesB		0.986	0.997	0.860
BayesCπ			0.976	0.812
TABLUP				0.876

### Accuracies and biases of GEBVs

The availability of true breeding values (TBVs) of the 1000 progenies without phenotypic values allowed a more efficient assessment for methods. Table 4 shows the correlations of TBVs and GEBVs, which measure the accuracies of GEBVs, and regressions of TBVs on GEBVs, which measure the biases of GEBVs, by different methods. In terms of both accuracy and bias, the three Bayesian methods and TABLUP performed similarly with correlations over 0.92 and slightly downward bias. BayesB and BayesCπ were slightly more accurate than BayesA and TABLUP, while TABLUP yielded smallest bias. GBLUP gave the lowest accuracy and the highest downward bias.

**Table 4:** Accuracies and biases of GEBVs for the 1000 progenies without phenotypic values.

Method	*r*	*b*
BayesA	0.924	1.063
BayesB	0.933	1.068
BayesCπ	0.938	1.057
TABLUP	0.924	1.033
GBLUP	0.774	1.648

*r*: correlation coefficient between GEBV and TBV; *b*: regression coefficient of TBV on GEBV.

TABLUP is an improvement of GBLUP in the way that the **G **matrix is replaced with **TA **matrix. In construction of the **TA **matrix, not only the marker genotypes, but also the marker effects are taken into account. The advantage of the **TA **matrix over the **G **matrix is that it not only accounts for the Mendelian sampling term, but also puts greater weight on loci explaining more of genetic variance for the trait of interest. This makes TABLUP more accurate than GBLUP. On the other hand, although TABLUP and the Bayesian methods gave similar accuracies, TABLUP has two important features that Bayesian methods lack. The first is that the reliability of an individual's GEBV can be calculated by TABLUP through the method outlined for GBLUP by VanRaden [3] and Strandén et al. [13]. The second is that TABLUP can be extended to estimate GEBVs for individuals without genotypes by constructing a joint pedigree-genomic relationship matrix according to the rule proposed by Legarra et al. [14].

## Conclusions

BayesA, BayesB, BayesCπ and TABLUP performed similarly and satisfactorily and remarkably outperformed GBLUP for genomic breeding value estimation in this dataset. TABLUP is a promising method for genomic breeding value estimation because of its easy computation of reliabilities of GEBVs and its easy extension to real life conditions such as multiple traits and consideration of individuals without genotypes.

## List of abbreviations used

QTL: quantitative trait locus; MAS: marker assisted selection; GS: genomic selection; BLUP: best linear unbiased prediction; GBLUP: BLUP with a realized relationship matrix; TABLUP: BLUP with a trait specific relationship matrix; EBV(s): estimated breeding value(s); GEBV(s): genomic estimated breeding value(s); TBV(s): true breeding value(s); SNP: single nucleotide polymorphism.

## Competing interests

The authors declare that they have no competing interests.

## Authors' contributions

CLW, PPM and ZZ contributed the data analyses and the manuscript. XDD and JFL contributed the modification of manuscript. WXF and ZQW carried out the data analyses. QZ coordinated the analyses and revised the manuscript. All authors have read and contributed to the final text of the manuscript.
